# Genetic Diversity and Population Structure of the USDA Sweetpotato (*Ipomoea batatas*) Germplasm Collections Using GBSpoly

**DOI:** 10.3389/fpls.2018.01166

**Published:** 2018-08-21

**Authors:** Phillip A. Wadl, Bode A. Olukolu, Sandra E. Branham, Robert L. Jarret, G. Craig Yencho, D. Michael Jackson

**Affiliations:** ^1^United States Vegetable Laboratory, United States Department of Agriculture, Agricultural Research Service, Charleston, SC, United States; ^2^Department of Horticultural Science, North Carolina State University, Raleigh, NC, United States; ^3^Department of Entomology and Plant Pathology, University of Tennessee, Knoxville, Knoxville, TN, United States; ^4^Plant Genetic Resources Conservation Unit, United States Department of Agriculture, Agricultural Research Service, Griffin, GA, United States

**Keywords:** Convolvulaceae, genotyping-by-sequencing, GBSpoly, polyploid, SNPs, sweetpotato, USDA germplasm

## Abstract

Sweetpotato (*Ipomoea batatas*) plays a critical role in food security and is the most important root crop worldwide following potatoes and cassava. In the United States (US), it is valued at over $700 million USD. There are two sweetpotato germplasm collections (Plant Genetic Resources Conservation Unit and US Vegetable Laboratory) maintained by the USDA, ARS for sweetpotato crop improvement. To date, no genome-wide assessment of genetic diversity within these collections has been reported in the published literature. In our study, population structure and genetic diversity of 417 USDA sweetpotato accessions originating from 8 broad geographical regions (Africa, Australia, Caribbean, Central America, Far East, North America, Pacific Islands, and South America) were determined using single nucleotide polymorphisms (SNPs) identified with a genotyping-by-sequencing (GBS) protocol, GBSpoly, optimized for highly heterozygous and polyploid species. Population structure using Bayesian clustering analyses (STRUCTURE) with 32,784 segregating SNPs grouped the accessions into four genetic groups and indicated a high degree of mixed ancestry. A neighbor-joining cladogram and principal components analysis based on a pairwise genetic distance matrix of the accessions supported the population structure analysis. Pairwise *F*_ST_ values between broad geographical regions based on the origin of accessions ranged from 0.017 (Far East – Pacific Islands) to 0.110 (Australia – South America) and supported the clustering of accessions based on genetic distance. The markers developed for use with this collection of accessions provide an important genomic resource for the sweetpotato community, and contribute to our understanding of the genetic diversity present within the US sweetpotato collection and the species.

## Introduction

Sweetpotato, *Ipomoea batatas* (L.) Lam. (Convolvulaceae), is the sixth most important food crop worldwide, following rice, wheat, potatoes, maize, and cassava (International Potato Center, 2018). This important root crop plays a critical role in food security, especially in developing countries. China is the largest producer of sweetpotato, accounting for over 70% of the world’s production, followed by Sub-Saharan Africa. While global sweetpotato production has been relatively stable for the past 45 years (Padmaja, 2009), production and consumption in the US has increased considerably since 2000 (United States Department of Agriculture, Economic Research Service [USDA-ERS], 2016). The US produced 3.1 billion tons of sweetpotatoes in 2015 and is ranked in the top 10 countries in annual worldwide production of the crop (United States Department of Agriculture, Economic Research Service [USDA-ERS], 2016). One of the reasons for increased production and consumption appears to be increased public awareness of the health benefits of sweetpotatoes and the production of a wide array of value-added products from the crop. Sweetpotatoes not only provide a source of carbohydrates, but are a major source of vitamins A (carotenoids from the orange-fleshed types), C, B1, B2 (riboflavin), B3 (niacin), B6, E, biotin, and pantothenic acid, as well as dietary fiber, potassium, copper, manganese, and iron; additionally, they are low in fat and cholesterol (Hill et al., 1992; Wang et al., 2016).

The origin of cultivated sweetpotato is unclear (Rajapakse et al., 2004; Roullier et al., 2013b). The most recent proposed origin of sweetpotato is of autopolyploid origin with *I. trifida* as the sole relative (Munoz-Rodriguez et al., 2018). Another hypothesis has proposed that *I. batatas* is an allo-autohexaploid (2*n* = 6*x* = 90), with a B_1_B_1_B_2_B_2_B_2_B_2_ genome composition resulting from an initial crossing between a tetraploid ancestor and a diploid progenitor followed by a whole genome duplication event (Magoon et al., 1970; Yang et al., 2016). It has also been proposed that sweetpotato originated from hybridization by unreduced gametes of diploid *I. trifida* and a tetraploid *I. batatas* (Shiotani, 1987; Orjeda et al., 1989; Oracion et al., 1990; Freyre et al., 1991), or that the species is derived from *I. trifida* and *I. triloba* (Austin, 1988).

Regardless of its origin, the sweetpotato genome is hexaploid and highly heterozygous, and this genetic complexity has slowed genome sequencing, assembly, and annotation over the past 10 years. Nevertheless, the available molecular resources for sweetpotato are rapidly expanding and include *de novo* assembled transcriptomes of sweetpotato and several of its predicted wild-type relatives (Schafleitner et al., 2010; Wang et al., 2010; Tao et al., 2012; Xie et al., 2012; Effendy et al., 2013; Firon et al., 2013; Solis et al., 2014, 2016; Ponniah et al., 2017), microsatellite, specific length amplified fragment (SLAF) and amplified fragment length polymorphism (AFLP) markers to characterize genetic diversity (Bruckner, 2004; Techen et al., 2009; Schafleitner et al., 2010; Roullier et al., 2011, 2013a,b; Su et al., 2017), recently released draft genome assemblies (Yang et al., 2016; Zhou et al., 2017) and whole chloroplast genomes of sweetpotato and wild relatives (Munoz-Rodriguez et al., 2018). Diploid reference genome assemblies based on progenitor wild relatives, *I. trifida* and *I. triloba*, are now available for hexaploid *I. batatas*. Assembled genomes together with gene annotations (32,301 annotated high confidence gene models of *I. trifida* and 31,426 of *I. triloba*) and aligned RNAseq data is available via the Genomic Tools for Sweetpotato Improvement Project, Sweetpotato Genomics Resource at Michigan State University (2018).

Genetic improvement of sweetpotato through traditional plant breeding is difficult due to its polyploid nature, genetic complexity, and high variability with regard to flower production and incompatibility (Jones et al., 1986). Generating additional (and leveraging existing) genomic resources would aid efforts to identify the molecular basis of phenotypic variation and advance the design of efficient and effective marker-assisted breeding strategies (Yoon et al., 2015). Marker-assisted breeding allows assessment of young plants at the seedling stage for multiple traits of interest, greatly reducing costs associated with growing the plants to maturity. This approach is especially valuable for sweetpotato, where the expense of long-term field evaluation is a major limiting factor in breeding efforts and where it is not feasible to conduct backcrossing breeding to introgress simple or oligogenic traits. Furthermore, genomic data provide a foundation to elucidate genetic relationships among parental lines and potentially identify new sources of genetic variation associated with environmental tolerance, pest and disease resistance, and other high-value traits. Genomic selection may also facilitate the assessment of hardiness, resistance to emerging diseases and insect pests, and changing consumer preferences. Inexpensive genome sequencing, innovative methods for the construction of nucleic acid libraries, improved mapping methodologies, and advanced computational approaches make genomics-based breeding a very attractive and powerful option for the improvement of sweetpotato.

The US sweetpotato germplasm collection is maintained by the USDA, ARS, Plant Genetic Resources Conservation Unit (PGRCU) in Griffin, Georgia, United States. This genebank maintains a diverse collection of *Ipomoea* spp. and provides clonal propagules of sweetpotato that are maintained as *in vitro* cultures. Available clones of hexaploid *I. batatas* were acquired over many decades, often in collaboration with various national and international programs and organizations. The collection provides the genetic foundation that supports ongoing research in breeding and genetics programs for the improvement of sweetpotato.

The USDA sweetpotato breeding program was initiated more than 45 years ago with the goal of developing germplasm resistant to soil insect pests while maintaining good horticultural characteristics. The germplasm used for improvement is primarily from materials developed and maintained at the USDA, ARS, US Vegetable Laboratory (USVL) in Charleston, South Carolina, United States and secondarily from the PGCRU collection. In general, resistance to insects is not associated with undesirable root quality traits in sweetpotato (Jones and Cuthbert, 1973). The program is based on recurrent mass selection using an open polycross system of 15-25 parental lines, and it relies on natural populations of bees for cross-pollination (Jones et al., 1986). To ensure that plant breeders continue to develop improved germplasm, there is a need for comprehensively phenotyped and genotyped germplasm collections. There has recently been significant progress in the phenotyping of storage root, foliage, and growth characteristics for over 700 accessions in the PGCRU collection (Jackson et al., 2018). There appears to be ample phenotypic diversity for root and vegetative phenotypic characteristics within the PGCRU and USVL collections, but there is a lack of knowledge with regard to the level of genetic diversity within both collections.

The objective of our study was to provide information on the level of genetic diversity contained within the combined USDA (PGRCU and USVL) sweetpotato collections. A major obstacle to utilizing the recently developed genomics resources for sweetpotato and other polyploid crops has been the lack of bioinformatics tools designed specifically to handle polyploid genetic data. Here, we take advantage of recently developed resources for polyploid genotyping and analysis at the genomic level. GBSpoly, an optimized genotyping-by-sequencing protocol for highly heterozygous and polyploid genomes, and GBSapp, a SNP calling and filtering bioinformatics pipeline, were used to identify 32,784 segregating SNP markers within a collection of 417 accessions from the combined collections. The markers developed for the analysis of this collection of accessions will provide an important genomic resource to the sweetpotato community.

## Materials and Methods

### Plant Materials

A total of 417 *Ipomoea batatas* accessions randomly selected from the PGCRU and USVL were examined in this study. Of the 417 accessions, 303 were from PGRCU and 114 accessions from USVL (**Table 1** and **Supplementary Table S1**). Eleven cultivars recommended for production in the southeastern US were part of the set of accessions, including Beauregard, Bayou Belle, Bellevue, Bonita, Burgundy, Carolina Ruby, Hayman White, Hernandez, Jewel, O’Henry, and Orleans. These materials originated from over 30 countries in 8 broad geographical regions (Africa, Australia, Caribbean, Central America, Far East, North America, Pacific Islands, and South America). Accessions were planted in field plots at the USVL and phenotyped for percent dry weight, periderm color, and stele color according to the methods of Jackson et al. (2018). Fresh leaf tissue was collected, placed into a labeled Ziploc^®^ style bag, stored on ice during the field collection process, and then immediately freeze-dried. Freeze-dried leaf tissue was stored at -20°C until used.

**Table 1:** Collection location of 417 sweetpotato (*Ipomoea batatas*) genotypes analyzed using GBSPoly.

Region (no. of accessions)	Countries (no. of accessions)
Africa (*n* = 15)	Nigeria (*n* = 14), Uganda (*n* = 1)
Australia (*n* = 2)	Australia (*n* = 2)
Caribbean (*n* = 18)	Cuba (*n* = 4), Puerto Rico (*n* = 14)
Central America (*n* = 26)	Costa Rica (*n* = 1), Guatemala (*n* = 20), Mexico (*n* = 5)
Far East (*n* = 47)	China (*n* = 16), Indonesia (*n* = 1), Japan (*n* = 11), Korea (*n* = 2), Philippines (*n* = 5), Taiwan (*n* = 10), Thailand (*n* = 1), Vietnam (*n* = 1), Unknown (*n* = 11)
North America (*n* = 210)	Canada (*n* = 1), United States (*n* = 209)
Pacific Islands (*n* = 42)	Cook Islands (*n* = 1), Fiji (*n* = 1), New Caledonia (*n* = 1), New Zealand (*n* = 12), Northern Mariana Islands (*n* = 1), Papua New Guinea (*n* = 17), Samoa (*n* = 4), Solomon Islands (*n* = 5)
South America (*n* = 44)	Brazil (*n* = 1), Columbia (*n* = 1), Ecuador (*n* = 1), Peru (*n* = 34), Uruguay (*n* = 4), Venezuela (*n* = 3)

### Genotyping, SNP Calling, and Dosage Calling

Total genomic DNA was isolated from freeze dried leaf tissue using the DNeasy Plant Mini Kit (Qiagen). The integrity, purity, and concentration of the isolated genomic DNA was determined by 2% agarose gel electrophoresis and a NanoDrop 2000 spectrophotometer (ThermoFisher). A modified genotyping-by-sequencing (GBSpoly) protocol optimized for highly heterozygous and polyploid genomes was implemented (Sweetpotato Genomics Resource at Michigan State University, 2018). A complementary bioinformatic pipeline, GBSapp, was used for SNP/dosage calling and data filtering. DNA concentrations were adjusted to 50 ng/μl. A double-digest was performed using 1 μg of DNA in a total volume of 30 μl with 5 units of *Cvi*AII at 25°C for 3 h and then with 5 units of *Tse*I at 65°C for 3 h in NEB CutSmart buffer (New England Biolabs). The digested DNA samples were purified with AMPure XP magnetic beads (ThermoFisher), quantified using a picogreen assay and then diluted to a concentration of 10 ng/μl. The resulting fragments were ligated to barcoded adapters which were designed to contain an 8-bp buffer sequence positioned upstream of the variable length (6–9 bp) barcode sequence (multiplexed for 96 pooled samples). Barcode design accounted for substitution and indel errors using the levenshtein/edit distance metric (Faircloth and Glenn, 2012). The buffer sequence ensures that the barcode sequence lies within a high-quality base call region of the sequence read. Aliquots of the samples were pooled and then a secondary double-digest with *Cvi*AII and *Tse*I (same enzymes and reactions conditions above) was performed to eliminate chimeric sequence ligations. The pools were again purified with AMPure XP magnetic beads and size-selected for 300–400 bp fragments using the Blue Pippin Prep system (Sage Science). PCR amplifications were performed (18 cycles) using NEB Phusion high-fidelity polymerase (New England Biolabs). The resulting libraries were size-selected again and then sequenced on an Illumina HiSeq 2500 system.

Raw Fastq files were processed by the following steps within the GBSapp pipeline, which integrates various software tools. The steps within the pipeline included:

(i) Using the FASTx-Toolkit^1^, QC plots of the raw reads were generated to ensure that the base calls within the barcodes had high quality scores.
(ii) Using the FASTx-Toolkit, the buffer sequence and any base position with low quality scores at the proximal end of the reads were trimmed to a quality score of at least Q36 for the lower whisker (minimum) in the boxplot (i.e., approximately 99.99% base calling accuracy, **Supplementary Figure S1**).
(iii) Demultiplexing of samples was performed with the FASTX-Toolkit.
(iv) Using BWA-MEM (Li, 2013), reads derived from each sample were mapped (using default parameters) to the two purported ancestral diploid reference genomes (*I. trifida* and *I. triloba*; Sweetpotato Genomics Resource at Michigan State University, 2018) of the hexaploid sweetpotato. Reads uniquely matching *I. trifida* and *I. triloba*, respectively, produced the 4x (tetraploid) and 2x (diploid) genotypes, while reads aligned to both genomes produced 6x (hexaploid) genotypes. On average, 90, 3.7, and 3.8% of the reads produced 6x, 4x, and 2x genotype calls, respectively.
(v) Additional processing of alignment files was performed with SAMtools (Li et al., 2009; Li, 2011) and Picard Tools^2^.
(vi) The GATK (version 3.7) HaplotypeCaller was used to call SNP, copy number variation (CNV), and Indel variants (McKenna et al., 2010; DePristo et al., 2011; Van der Auwera et al., 2013). Using VCFtools v.0.1.14 (Danecek et al., 2011), genotype calls and read depth information were extracted from the output VCF files for data filtering in R v 3.4.1 (R Core Team, 2012).
(vii) Data filtering in R was performed to identify high quality variants and bi-allelic variants. Thresholds were set for minor allele frequency (MAF) at 0.05 and missing data at no more than 20% missing. The optimal read depth for calling 2x (diploid), 4x (tetraploid), and 6x (hexaploid) genotype calls were empirically determined using a diagnostic tool written in R (R Core Team, 2012). This was accomplished by resampling (without replacement) reads from some individuals that were sequenced multiple times (replicated in multiplexed pools) to achieve very high read depth across all loci (i.e., as much as 500-2,000X coverage). Resampling was performed to capture 0.1% to 0.9% (increments of 0.1%) and 1–100% (increments of 1%) of the total reads. This simulated multiple passes of Illumina sequencing to capture each locus at various read depths. The stability of each genotype was measured across different read depths by comparing genotypes at all read depths for each locus to read depth at 100% resampling. At a 95% confidence limit, nulliplex (000000 or 111111) markers required a 1X-coverage threshold, simplex (000001 or 0111111) markers required a 35X-coverage threshold, while duplex (000011 or 0011111) and triplex (000111) markers required a 100X-coverage threshold. Aligning the reads underlying each variant back to each of the reference genomes revealed that the stable genotype calls were derived from unique sequences mapping to a single locus in the genome, while unstable genotype calls tended to map to multiple regions in the genome, suggesting that the sequence context was due to paralogs or repetitive sequences.

### Data Analysis

Marker summary statistics by broad geographical region, including allele frequencies and region-specific alleles, were calculated with SVS version 8.7.0 (Golden Helix). The markers were filtered by linkage disequilibrium to generate a set of unlinked SNPs to meet assumptions for population structure analyses in STRUCTURE. Linkage disequilibrium was calculated via the expectation-maximum method (Excoffier and Slatkin, 1995) using the LD prune function of SVS with the filtering parameters set to an *r*^2^ of 0.5, window size of 50, and window increment of 5. To determine the extent of LD decay in sweetpotato, LD was assessed by estimating *r*^2^ values (Hill and Robertson, 1968) for all marker pairs using R base, while a plot of LD (*r*^2^) against marker intervals (physical distance in bp) was implemented with ggplot2, a R package (R Core Team, 2012). The analyses were performed with the genotype data set that retained allelic dosage information as well as a diploidized genotype format The first five principle components of the reduced dataset were calculated in SVS using an additive model to visualize the genetic diversity across the collection (*N* = 417). To examine the population structure of sweetpotato accessions, they were clustered into populations with the program STRUCTURE v2.3.4 using the admixture model with correlated allele frequencies (Pritchard et al., 2000; Falush et al., 2003, 2007; Hubisz et al., 2009) using the set of 32,784 segregating SNPs. Population numbers (K) of 1 to 10 were run 10 times each with 35,000 burn-in iterations and 35,000 Markov Chain Monte Carlo repetitions. Estimation of the best *K* value was determined using STRUCTURE Harvester (Earl, 2012), which identifies the appropriate number of clusters (*k*) using the *ad hoc* statistic delta K (Evanno et al., 2005). This is based on the second order rate of change in the log probability of the data between successive values of *k*.

Pairwise genetic distance matrices using polymorphic markers were created in MEGA v6.06 with the *p*-distance model (Tamura et al., 2013). An unrooted neighbor joining phylogenetic tree was constructed in MEGA v6.06 (Tamura et al., 2013) using a pairwise genetic distance matrix of 417 accessions. The interior-branch test method (1,000 bootstrap replications) determined branch support and branches of less than 50% confidence were collapsed. FigTree v1.4.2 was used to transform the phylogenetic tree to a cladogram (Rambaut, 2014).

## Results

### SNP Calling and Allele Dose-Dependent Genotypes

The raw fastq files generated on Illumina HiSeq2500 platform were processed to evaluate the distribution of quality scores at each base position along the sequence reads (**Supplementary Figure S1**). The quality scores were empirically determined based on aligning reads to the Illumina’s PhiX control. All the bases within the barcode region and the genomic insert had a high quality score of 38 or approximately 99.984% accuracy (i.e., median, quartiles and minimum in boxplot), except for the last base call which had a minimum score of 28 and was trimmed off (first quartile ranging from 28 to 34, an inter-quartile range of 34–38, and a median of 38). This high accuracy in the base calls of the barcode sequence is particularly important for accurate de-multiplexing of the reads derived from pooled samples. The first 5–6 bp of this 8 bp buffer sequence had slightly lower quality score (32–34), while the next 2–3 base calls had a quality score of 38. These 8 bases were trimmed off before de-multiplexing.

After aligning the raw reads to the two ancestral reference sub-genomes, on average, 96.05% of the raw reads mapped to both reference genomes. On average, 85.7% of reads matched to both sub-genomes (genotypes with 6 alleles across 6 homeologous chromosomes), 3.1% were specific to the *I. trifida* subgenome (genotypes with 4 alleles across 4 of the 6 homeologous chromosomes), and 7.2% were specific to the *I. triloba* subgenome (genotypes with 2 alleles across 4 of the 6 homeologous chromosomes). Reads specific to the *I. triloba* subgenome tended to have higher proportions than those specific to *I. trifida*-specific (**Supplementary Figure S2**). Even though more subgenome specific reads matched *I. triloba*, the *I. trifida* produced more unfiltered SNPs (496,157 against 311,409). This suggests more reads specific to the *I. triloba* subgenome might be enriched and derived from repetitive sequences. Distribution of read depth was relatively uniformly distributed across the sweetpotato accessions and across genomic loci, except for a few individuals that were underrepresented in the library (**Supplementary Figure S3**).

**FIGURE 1:**
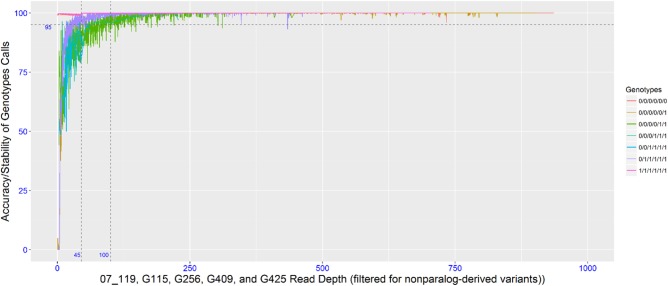
Plot showing read depth thresholds and the associate SNP calling accuracy (or genotype stability with varying read depth).

**FIGURE 2:**
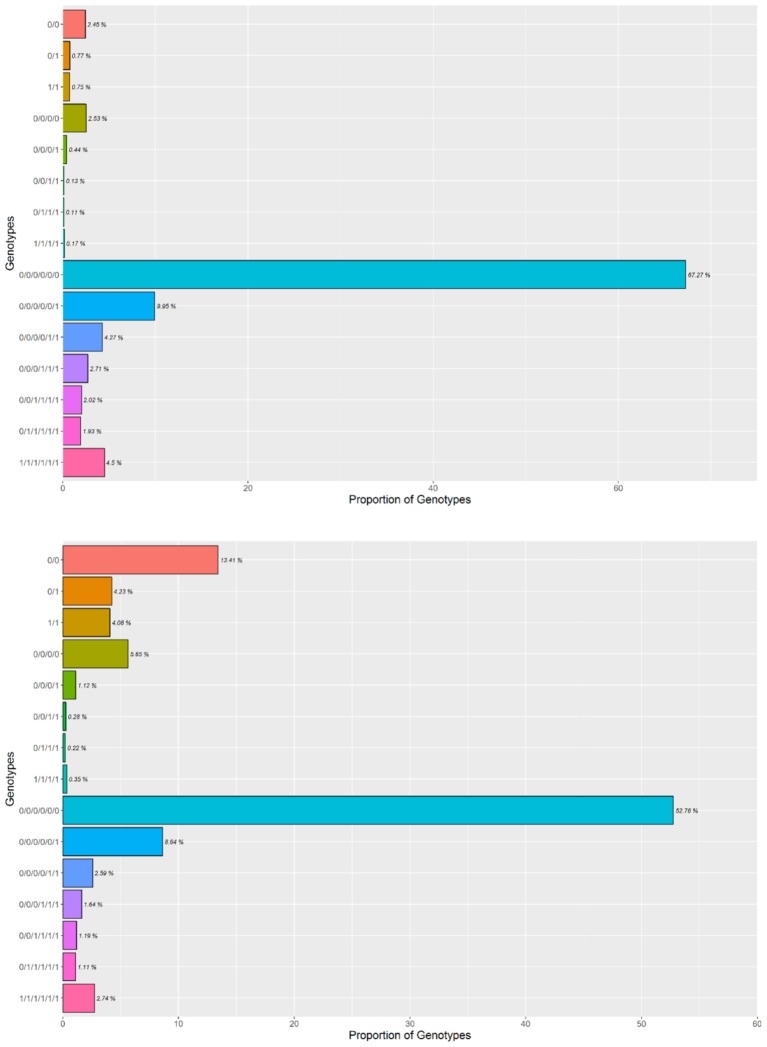
Proportions of dose-dependent genotype calls at two different read depth thresholds of 6, 20, and 45 for 2x, 4x, and 6x genotypes, respectively, **(top)** and 6, 35, and 100 for 2x, 4x, and 6x genotypes, respectively **(bottom)**.

To empirically determine the optimal read depth threshold for accurately calling the dose-dependent genotypes, four individuals with high sequencing coverage/read depth were resampled (as described in the methods). The accuracy (or dose-dependent genotype stability) associated with the genotypic classes was determined by this resampling method. To achieve a 95% accuracy in 6x genotypes, the read depth threshold required for simplex and multi-dose genotypes was 35 and 100, respectively, while an 85% accuracy required a read depth threshold of 20 and 45. Since the data set is comprised of different dose-dependent genotypic classes that were not known *a priori*, a read threshold of 45 was used. A read depth threshold of 45 indicates almost 100% accuracy for nulliplex genotypes, over 95% accuracy for simplex genotypes and about 85% accuracy for multi-dose genotypes (**Figure 1**). Most of the genotypes were nulliplex and simplex (**Figure 2**).

### Genetic Diversity and Population Structure in the Germplasm Accessions From the PGCRU and USVL

A total of 417 *I. batatas* accessions from 8 broad geographical regions (Africa, Australia, Caribbean, Central America, Far East, North America, Pacific Islands, and South America) were genotyped at 43,105 diploidized SNPs derived from GBSpoly (**Table 1** and **Supplementary Table S1**; SRA Accession SRP152827). Pruning by linkage disequilibrium resulted in a set of 32,784 segregating markers used for all analyses (**Supplementary Dataset S1**). Genetic distances between accessions (measured as the proportion of variant alleles) ranged from 0.035 to 0.41, with a mean of 0.31 and a standard deviation of 0.028 (**Supplementary Table S2**). The distribution of pairwise genetic distances had a narrow spread with only 0.1% of the comparisons less than 0.1 and 0.02% greater than or equal to 0.4. The cultivars (*n* = 11) recommended for production in the southeastern US had lower genetic diversity with a mean genetic distance of 0.25. The most similar cultivars, Bayou Belle and Burgundy, only differed at 4.5% of their alleles. Jewel was the most genetically distinct cultivar and was present in all of the top 10 most genetically distant pairwise comparisons within the cultivars (0.32–0.34). The remaining cultivar comparisons did not exceed 0.30. The overall mean MAF was 0.219 and ranged from 0.204 (South America) – 0.237 (Central America) by geographical regions (**Table 2**). Allele frequencies and Hardy-Weinberg equilibrium values for each SNP by region are provided in **Supplementary Table S3**.

**Table 2:** Minor allele frequency (MAF) for the 417 *Ipomoea batatas* accessions that were grouped by geographical region.

Broad geographical region (No. accessions)	Mean MAF	Range MAF
Africa (*n* = 15)	0.220	0–1.000
Australia (*n* = 2)	0.227	0–1.000
Caribbean (*n* = 18)	0.223	0–0.933
Central America (n = 26)	0.237	0–0.956
Far East (*n* = 55)	0.224	0–0.887
North America (*n* = 210)	0.211	0–0.730
Pacific Islands (*n* = 47)	0.221	0–0.930
South America (*n* = 44)	0.204	0–0.907
Total (*n* = 417)	0.219	0.041–0.050

**FIGURE 3:**
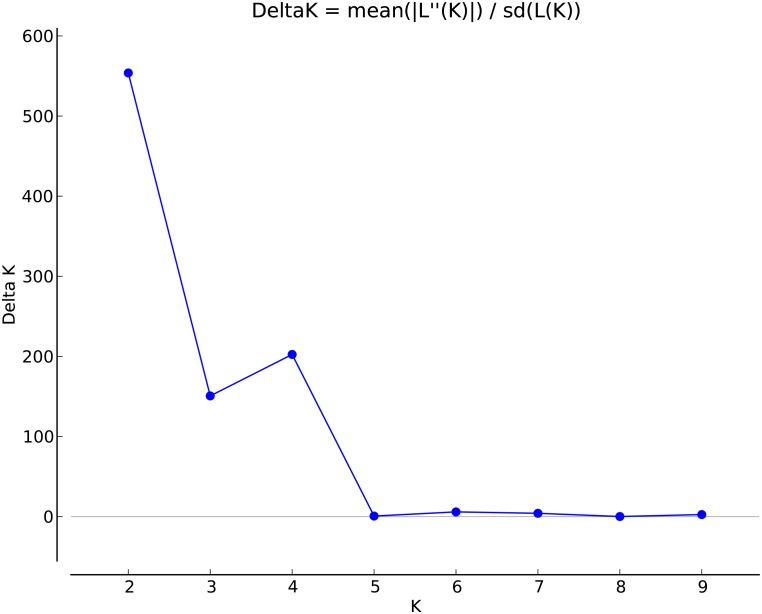
Delta *K* values for different numbers of populations (K) assumed in STRUCTURE analysis.

**FIGURE 4:**
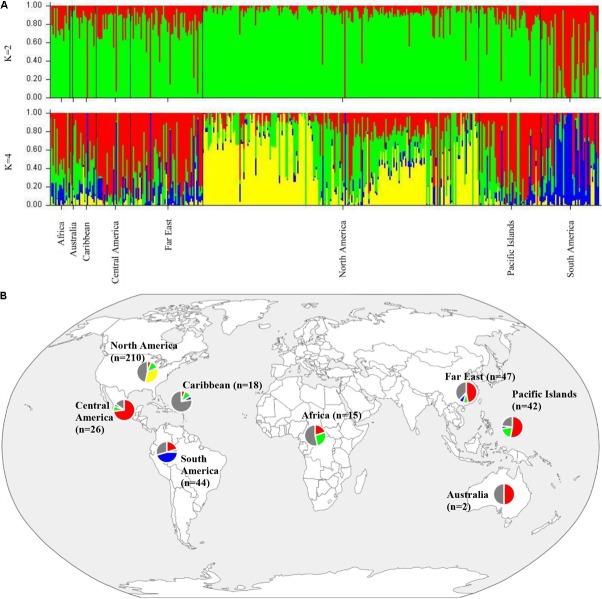
**(A)** Bar plots of Bayesian assignment probabilities for each *Ipomoea batatas* accession analyzed with 32,784 SNPs using the program STRUCTURE for 2 (*K* = 2) or 4 clusters (*K* = 4). The *x*-axis indicates accession and the y-axis indicates the assignment probability of that accession to each of the four clusters. Each vertical line represents an individual’s probability of belonging to one of K clusters (represented by different colors) or a combination of clusters if ancestry is mixed. **(B)** Map of the sampled regions for 417 *Ipomoea batatas* accessions. Pie charts correspond to the population assignment for the four genetic groups defined by the Bayesian assignment of STRUCTURE. Accessions were assigned to a cluster based on probabilities calculated in STRUCTURE, where C1 is red, C2 is green, C3 is blue, and C4 is yellow. A *q*-value threshold of 0.65 was used to divide the accessions into one of the four clusters or as admixed (gray section of pie charts).

To determine population structure, STRUCTURE 2.3.4 was used. The largest delta *K* peak was observed at *K* = 2 and a smaller peak was seen at *K* = 4 (**Figure 3**). We adopted the grouping for *K* = 4, because this represents a more accurate estimate of the gene pools given the historical movement of sweetpotato germplasm and that developed through crop improvement programs. There was a high degree of admixture observed in cluster assignments of the accessions with both population numbers but it was higher for *K* = 4 (**Figure 4**). When *K* = 2, the accessions clustered into two groups (South America and the other regions, **Figure 4B**). Alternatively, when *K* = 4 is considered, there is a much higher degree of admixture and assignment of accessions to clusters into four gene pools (**Figure 4**). Despite the high degree of admixture within the accessions, our analyses supported dividing the 417 PIs into four clusters using a *q*-value threshold of 0.65 (**Figure 2** and **Supplementary Figure S4**), where C1 is red, C2 is green, C3 is blue, C4 is yellow, and gray is admixed (**Figure 4** and **Supplementary Figure S4**). In total, 247 out of 417 accessions (60%) were assigned to 1 of the 4 clusters. Clusters 1 through 4 consisted of 96 (23%), 49 (12%), 29 (7%), and 73 (18%) accessions, respectively. The remaining 170 accessions (40%) were categorized as having admixed ancestry from the clusters (**Supplementary Table S1**). Cluster 1 consisted mostly of accessions from Central America (*n* = 19), the Far East (*n* = 26), and the Pacific Islands (*n* = 25); cluster 2 from North America (*n* = 26) and the Pacific Islands (*n* = 9); cluster 3 from South America (*n* = 20); and cluster 4 consisted only of accessions from the North America [United States (*n* = 73)]. Accessions with mixed ancestry were found from all the studied regions: Africa (53.3%), Australia (50%), Caribbean (77.8%), Central America (15.4%), Far East (44.7%), North America (46.7%), Pacific Islands (26.2%), and South America (29.6%).

**FIGURE 5:**
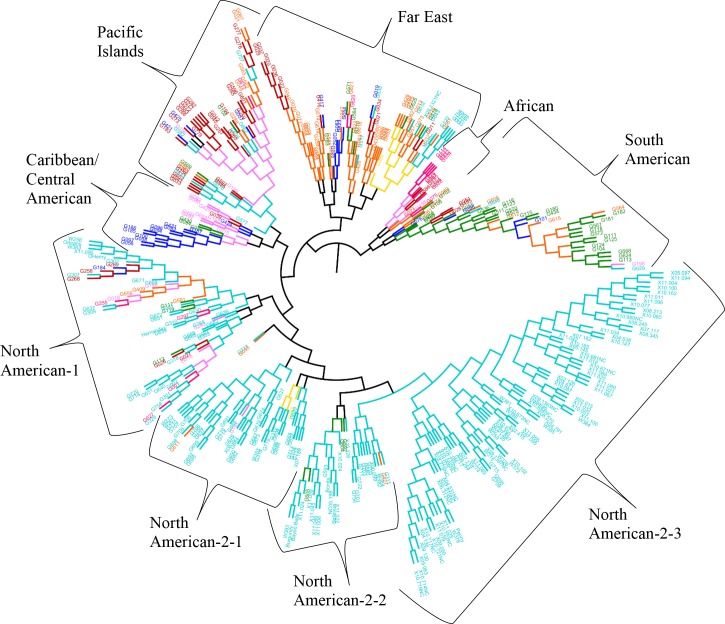
Neighbor-joining tree based on genetic distances for 417 *Ipomoea batatas* accessions from 8 geographical regions using 32,784 single nucleotide polymorphisms. The interior-branch test method (1,000 bootstrap replications) determined branch support and branches of less than 50% confidence were collapsed. Each accession is colored according to their geographical origin, with the RGB color value used in FigTree listed in parentheses. Africa = pink (F70A7B), Australia = yellow (F7DA10), Caribbean = purple (F77AF8), Central America = blue (030EF8), Far East = orange (F96509), North America = turquoise (00CCCC), Pacific Islands = red (BC0302), South America = green (0F890E).

**FIGURE 6:**
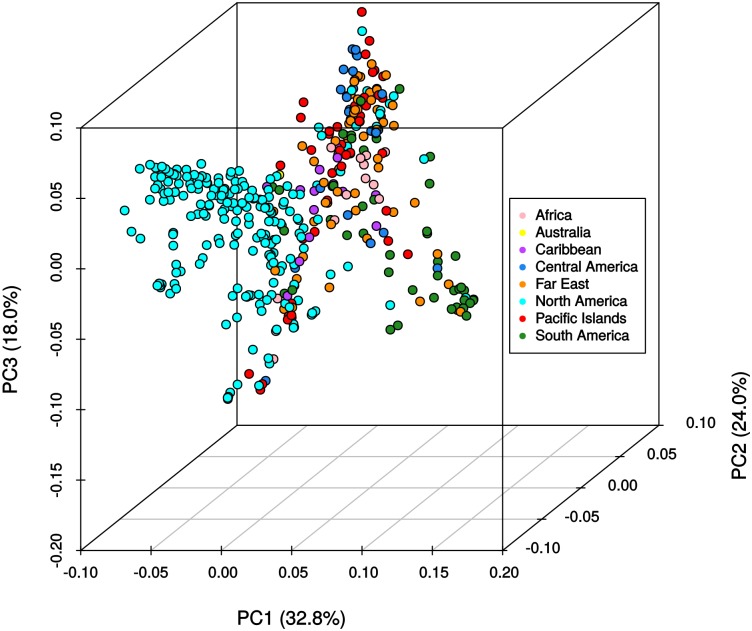
Principle components analysis of 417 *Ipomoea batatas* accessions using 32,784 single nucleotide polymorphisms. The percent variation explained by each principle component is indicated in parentheses. Geographical regions are color-coded according to the legend.

The results from the Bayesian clustering method (STRUCTURE) were further supported by population differentiation and genetic diversity analyses. Population differentiation, measured as pairwise *F*_ST_ values between geographical regions (**Table 3**), supported the cluster assignments for *K* = 4. The greatest differentiation was between South American and other regional groups, as all values were ≥ 0.063 and ranged from 0.063 (Far East) to 0.110 (Australia). Values differentiating the North American group were the greatest between it and the South American (0.103) and Central American (0.074) groups and the lowest between it and the Caribbean group (0.031). In general, differentiation was low between regions, as 17 of the 28 pairwise *F*_ST_ values were ≤ 0.05. The lowest differentiation was between the Far East and Pacific Islands (0.017) and the Far East and Australia (0.018). Further corroboration for the Bayesian clustering method was observed in the neighbor-joining (NJ) cluster analysis (**Figure 5**) and the principal components analysis [PCA (**Figure 6**)]. The NJ cladogram clearly separated the accessions into four groups, primarily based on region of collection (North American 1 and 2, South American, and the remaining regions). The PCA also separated the accessions into four groups and was consistent with the individual assignments made with STRUCTURE (**Figure 4** and **Supplementary Figure S4**). There was no apparent pattern of the accessions in the PCA by dry weight or periderm (skin) color, but there was clustering by stele (flesh) color (**Supplementary Figure S5**).

**Table 3:** Pairwise *F*_ST_ values between broad geographical regions based on the collection location of *Ipomoea batatas* accessions.

	Africa	Australia	Caribbean	Central America	Far East	North America	Pacific Islands	South America
Africa	0.000							
Australia	0.050	0.000						
Caribbean	0.021	0.035	0.000					
Central America	0.054	0.033	0.039	0.000				
Far East	0.032	0.018	0.031	0.033	0.000			
North America	0.056	0.049	0.043	0.074	0.052	0.000		
Pacific Islands	0.031	0.030	0.026	0.034	0.017	0.050	0.000	
South America	0.084	0.110	0.068	0.093	0.063	0.103	0.087	0.000

### Linkage Disequilibrium in Sweetpotato

Knowing the extent of linkage disequilibrium decay in a crop is crucial for determining if genome-wide association analysis in diverse germplasm can be used for fine-mapping QTL to genic resolution. We estimated *r*^2^ values for all marker pairs within and between chromosomes in order to empirically determine LD decay. LD analysis based on the 417 sweetpotato accessions revealed that linkage disequilibrium blocks (LD decays between 0.6 and 1.2 kb at a *r*^2^ threshold of 0.1 and 0.2, respectively) are small enough for fine-mapping to the gene-level (**Supplementary Figure S6**). Genotypes with allelic dosage information and the diploidized genotype data set both revealed very similar trends.

## Discussion

Sweetpotato is widely produced throughout the tropical regions of the world and plays a critical role in food security. Within the US, increased consumption and exports of sweetpotato have spurred substantial economic growth for producers as the value of the crop has increased by over $500 million USD between 2000 to 2015 (United States Department of Agriculture, Economic Research Service [USDA-ERS], 2016). The US sweetpotato germplasm collections maintained by the PGRCU and USVL have a general lack of genetic information, which poses challenges for germplasm curators, breeders and geneticists, entomologists, horticulturalists, and plant pathologists. In our study, two US sweetpotato germplasm collections (PGCRU and USVL) were genotyped with 32,784 SNPs to characterize genetic diversity and population structure. We found that genetic diversity and population structure were associated with geographic region and that collections had high levels of mixed ancestry (40%).

Our analyses are the first to report the use of SNPs to characterize US sweetpotato germplasm collections and the second reported for any sweetpotato collection. Su et al. (2017) assessed diversity and population structure of 197 accessions that were mostly from China (*n* = 178) and found high levels of genetic diversity and support for 3 groups. Our mean minor allele frequency (0.219) was similar to their reported value (0.216). We were unable to compare the degree of admixture as it was not discussed by Su et al. (2017). However, we found a few instances of clustering of accessions into groups that did not match the geographic collection location. This can likely be explained as the result of exchange of germplasm between regions and/or incomplete or inaccurate information about these accessions in the germplasm database.

Molecular markers (AFLPs and RAPDs) have been used to examine the diversity of sweetpotato in Oceania (Zhang et al., 1998, 2004; Gichuki et al., 2003; Bruckner, 2004). These studies demonstrated that materials from the Pacific were more genetically related to materials from Mesoamerica (Mexico). More recently, chloroplast and nuclear microsatellite markers were used to demonstrate the existence of a northern and a southern gene pool of sweetpotato from Tropical America (Roullier et al., 2011). The northern gene pool was composed of materials from the Caribbean and Central America, whereas the materials in the southern gene pool were from the Peru-Ecuador region of South America. Munoz-Rodriguez et al. (2018) using whole chloroplast genomes, demonstrated the existence of two distinct sweetpotato lineages that supported the findings of Roullier et al. (2013a) using chloroplast microsatellites. Our results support the existence of multiple gene pools within the USDA sweetpotato collections. The least admixture was seen in accessions from Central America and South America and appears to support the occurrence of a northern and a southern gene pool as indicated by Roullier et al. (2011) and Munoz-Rodriguez et al. (2018). Additionally, our findings provide evidence to support the *K* = 3 that was reported by Roullier et al. (2013a, Figure 2 in Appendix S1) in the sweetpotato clones from tropical America. We obtained similar results for accessions collected from this region when Bayesian clustering methods were used. We also provide evidence for a third gene pool within our samples from tropical America (**Figure 4** and **Supplementary Figure S4**, green cluster). The existence of a third gene pool would account for the subset of accessions not developed by the USVL that were clustered within the materials from North America. The majority of the accessions from the Far East (55%) have probability assignments in STRUCTURE similar to the Central American accessions (**Supplementary Table S1**, **Figure 4**, and **Supplementary Figure S4**). We speculate that this third gene pool from tropical America (Roullier et al., 2011) is the source of genetic material that has been introgressed into US sweetpotato accessions and that can be traced to the cultivar ‘Porto Rico’ (individual #234 in **Supplementary Figure S4**, PI 566646). ‘Porto Rico’ was imported into the US from Puerto Rico in 1906 and the sweetpotato industry was subsequently built around it (Harmon et al., 1970). Additional accessions that have been used in the development of the North American germplasm are PIs 153655, 208029, 399163, 399164, 153907, 296116, 344124, 566636, 286619, 286621, 308196, 318848, 318855, 318858, 324885, and 344140 (Harmon et al., 1970; Jones et al., 1991, **Supplementary Table S1** and **Supplementary Figure S4**) and introgression of these are evident within the North American accessions. We also found support of a second gene pool within the North American accessions (**Figure 4** and **Supplementary Figure S4**). We suspect that this gene pool is the result of the heavy selection pressure that has been used in the development of this germplasm (USVL collection). The W-lines and cultivars developed by the USDA sweetpotato breeding program are the result of mass selection using parents of diverse origins and this is reflected in the high level of mixed ancestry within these materials (75%). These individuals were selected for multiple insect, nematode, and disease resistance traits in combination with other desirable production and market quality traits (Jones et al., 1991). We suspect that the USVL-lines (**Supplementary Table S1** and **Supplementary Figure S4**) show a less diverse genetic base due to this material being developed through recurrent selection for insect resistance where the most resistant selections were used as parents in subsequent breeding cycles as compared to the mass selection techniques used in the development of the USVL W-lines (Jones and Dukes, 1976; Jones et al., 1986, 1991). Within the group of USVL-Lines, the introgression of material from other gene pools is evident. For example, individuals with STRUCTURE IDs 32, 41, 59, 64, 65, 70, 71, 72, and 73 in **Supplementary Figure S4** can trace their ancestry to the Uruguayan and the Louisiana State University sweetpotato breeding programs as material from these programs were used as parents in crossing blocks. Two individuals (29 and 79, **Supplementary Figure S4**) exhibit a significantly different background than the other USVL-lines. We believe that this is due to incomplete or erroneous information regarding the origins of the parents of those lines.

Germplasm collections are critical for providing genetic materials needed to ensure a continued global supply of food. Plant breeding and the associated disciplines require well characterized and readily available germplasm resources to develop crops with resistance/tolerance to pests, disease, and environmental stress. Our results indicate that there is high genetic diversity within the US sweetpotato collection and now there is the potential to utilize genotype data from our study and corresponding phenotype data (Jackson et al., 2018) for selection of a core germplasm collection. The markers developed for use with this collection of accessions provide an important genomic resource for the sweetpotato community and contribute to our understanding of the genetic diversity present in the US sweetpotato germplasm.

## Author Contributions

PW, BO, SB, RJ, GY, and DJ: conceived and designed the experiments. PW, BO, and SB: performed the experiments. PW, BO, SB, RJ, GY, and DJ: analyzed the data. PW, BO, SB, RJ, and GY: contributed reagents, materials, and analysis tools. PW, BO, and SB: wrote the paper. PW, BO, SB, RJ, GY, and DJ: read and approved the manuscript. PW, BO, and SB: contributed equally to this project.

## Conflict of Interest Statement

The authors declare that the research was conducted in the absence of any commercial or financial relationships that could be construed as a potential conflict of interest.

## Footnotes

1 http://hannonlab.cshl.edu/fastx_toolkit/

2 https://broadinstitute.github.io/picard/

## Supplementary Material

FIGURE S1 QC Boxplot showing distribution of quality scores of raw reads in a multiplexed library containing 96 *Ipomoea batatas* accessions. Buffer sequence lie within the first 8 base calls, while variable barcodes (6–9 bp) lie at position 14–17 bp.

FIGURE S2 Proportion raw reads matching both reference subgenomes (6x genotypes) and those specific to each of the subgenomes (4x and 2x genotypes derived *Ipomoea trifida* and *I. triloba*, respectively).

FIGURE S3 Boxplot shows relatively uniform read depth across individual samples and genomic loci after de-multiplexing pool samples. Only genotypes with 6 alleles/dose are shown here.

FIGURE S4 Bar plots of Bayesian assignment probabilities for each *Ipomoea batatas* accession analyzed with segregating 32,784 SNPs using the program STRUCTURE for *K* = 4. The *x*-axis indicates accession and the *y*-axis indicates the assignment probability of that accession to each of the four clusters. Each vertical line represents an individual’s probability of belonging to one of K clusters (represented by different colors) or a combination of if ancestry is mixed. The asterisk (^∗^) indicates the cultivar Porto Rico, which is a foundational line of the sweetpotato industry in the US. The plus sign (+) indicates that this accession was used as parental material in the mass selection populations developed by Jones et al. (1991). The USDA, ARS, US Vegetable Laboratory (USVL) W-lines and USVL-lines originate from the mass selection populations. Information for all accessions is found in **Supplementary Table S1**.

FIGURE S5 Linkage disequilibrium estimates (*r*^2^) of all genome-wide marker pairs plotted against corresponding interval between marker pairs. Curve (blue line) based on game smoothing method function shows distribution of all data points. Top and middle plot based on genotype data with allelic dosage information, while bottom plot is based on diploidized genotypes.

TABLE S1 Information of *Ipomoea batatas* accessions analyzed by GBSpoly.

TABLE S2 Pairwise genetic distance matrix between *Ipomoea batatas* accessions.

TABLE S3 Information for individual SNPs used for data analyses.

DATASET S1 Structure data file for *Ipomoea batatas* accessions.
