# Supplementary figures and images for: Genetic Diversity and Population Structure of the USDA Sweetpotato (Ipomoea batatas) Germplasm Collections Using GBSpoly
Source: Front Plant Sci. 2018 Aug 21;9:1166. doi: 10.3389/fpls.2018.01166 (PMC6111789; doi:10.3389/fpls.2018.01166)

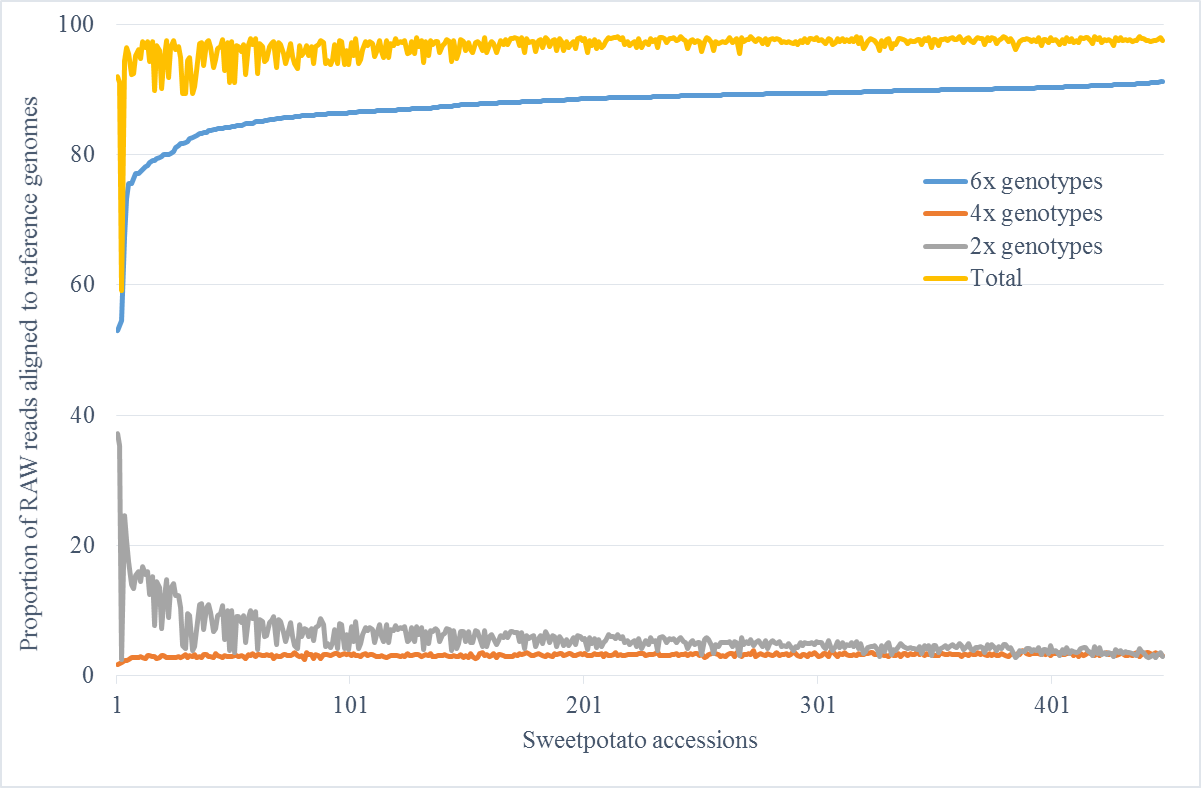

Supplement: FIGURE S2 — Proportion raw reads matching both reference subgenomes (6x genotypes) and those specific to each of the subgenomes (4x and 2x genotypes derived Ipomoea trifida and I. triloba, respectively). [file Image_2.TIF]

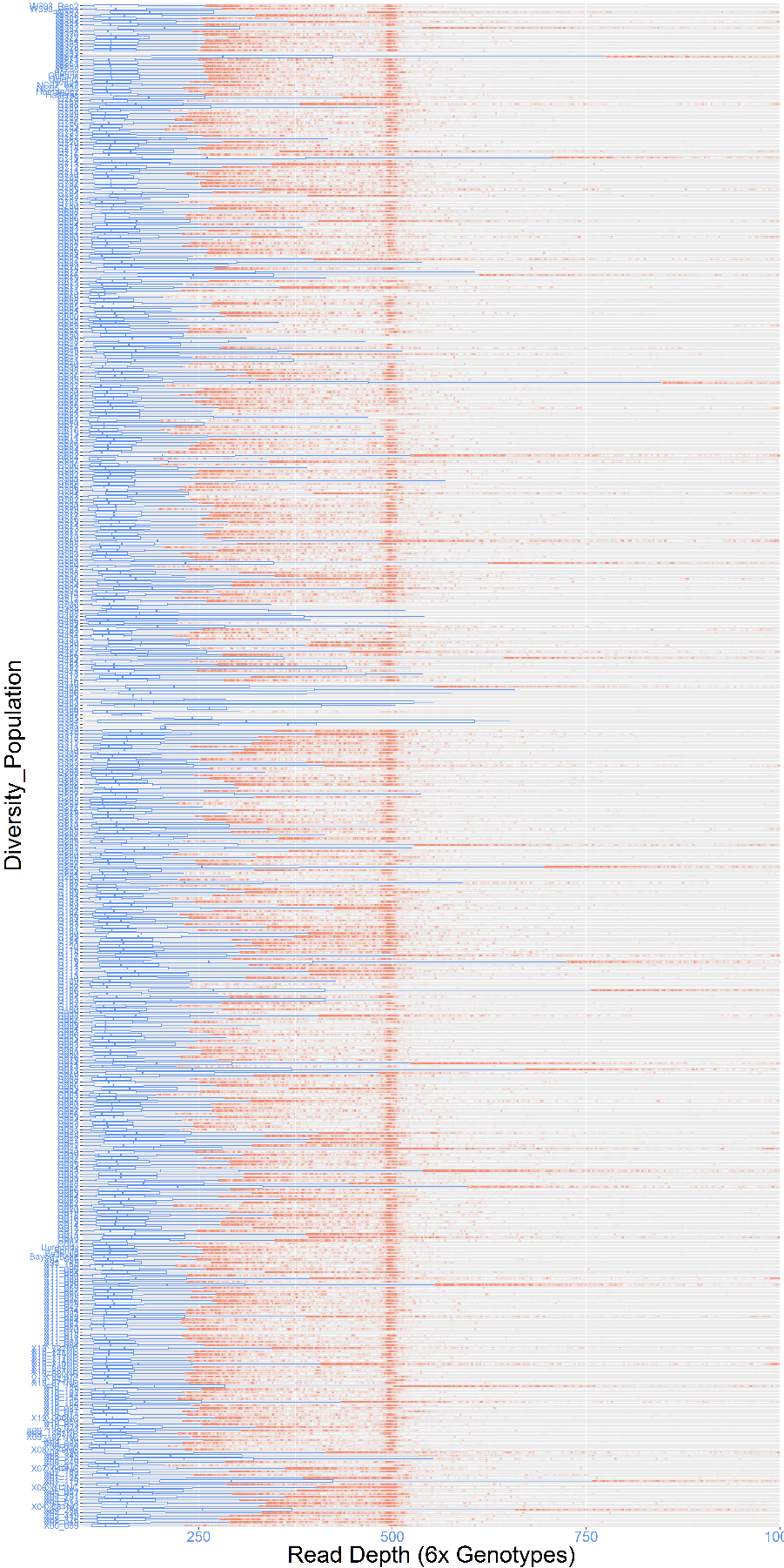

Supplement: FIGURE S3 — Boxplot shows relatively uniform read depth across individual samples and genomic loci after de-multiplexing pool samples. Only genotypes with 6 alleles/dose are shown here. [file Image_3.TIF]

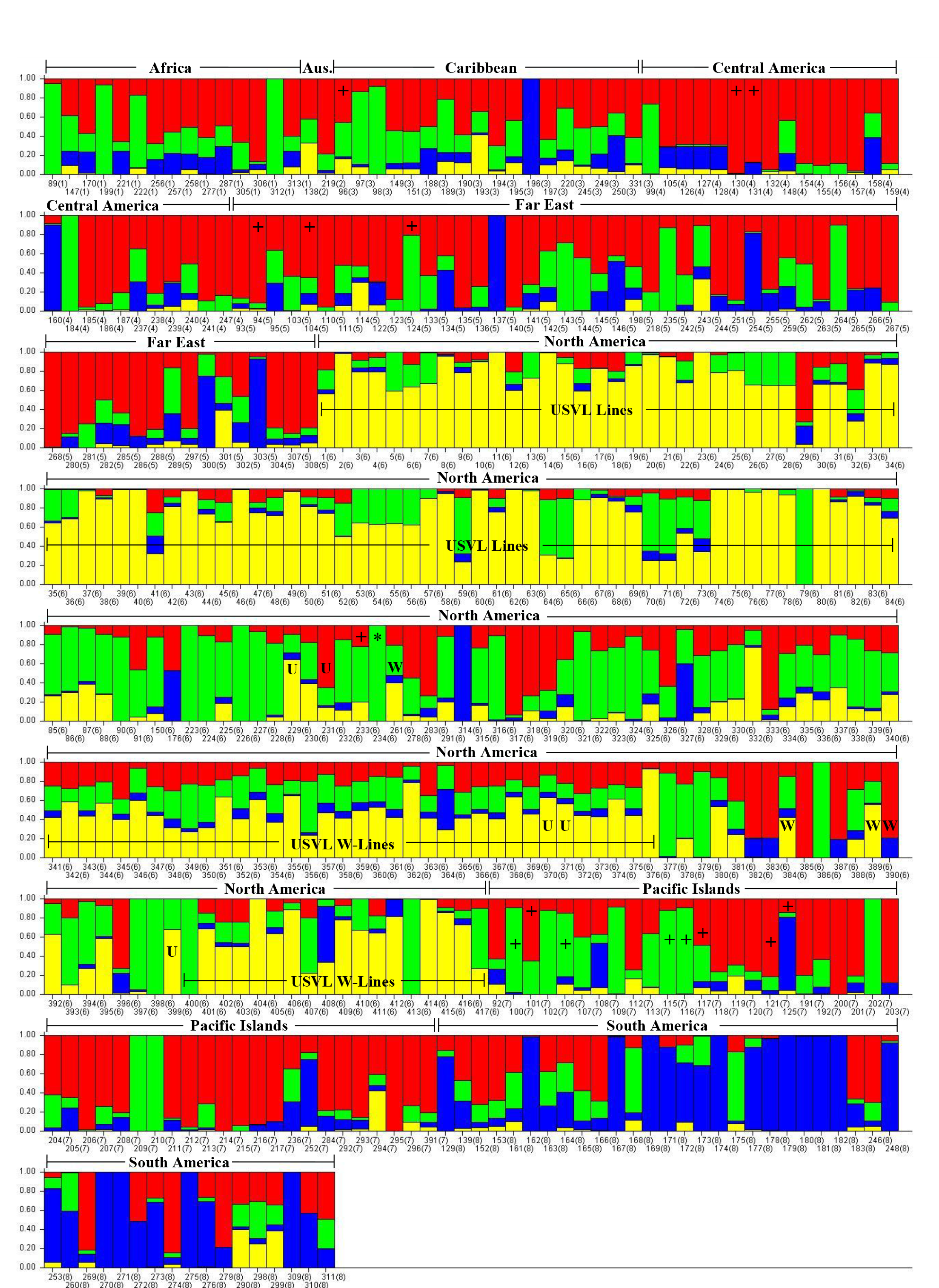

Supplement: FIGURE S4 — Bar plots of Bayesian assignment probabilities for each Ipomoea batatas accession analyzed with segregating 32,784 SNPs using the program STRUCTURE for K = 4. The x-axis indicates accession and the y-axis indicates the assignment probability of that accession to each of the four clusters. Each vertical line represents an individual’s probability of belonging to one of K clusters (represented by different colors) or a combination of if ancestry is mixed. The asterisk (∗) indicates the cultivar Porto Rico, which is a foundational line of the sweetpotato industry in the US. The plus sign (+) indicates that this accession was used as parental material in the mass selection populations developed by Jones et al. (1991). The USDA, ARS, US Vegetable Laboratory (USVL) W-lines and USVL-lines originate from the mass selection populations. Information for all accessions is found in Supplementary Table S1. [file Image_4.TIF]

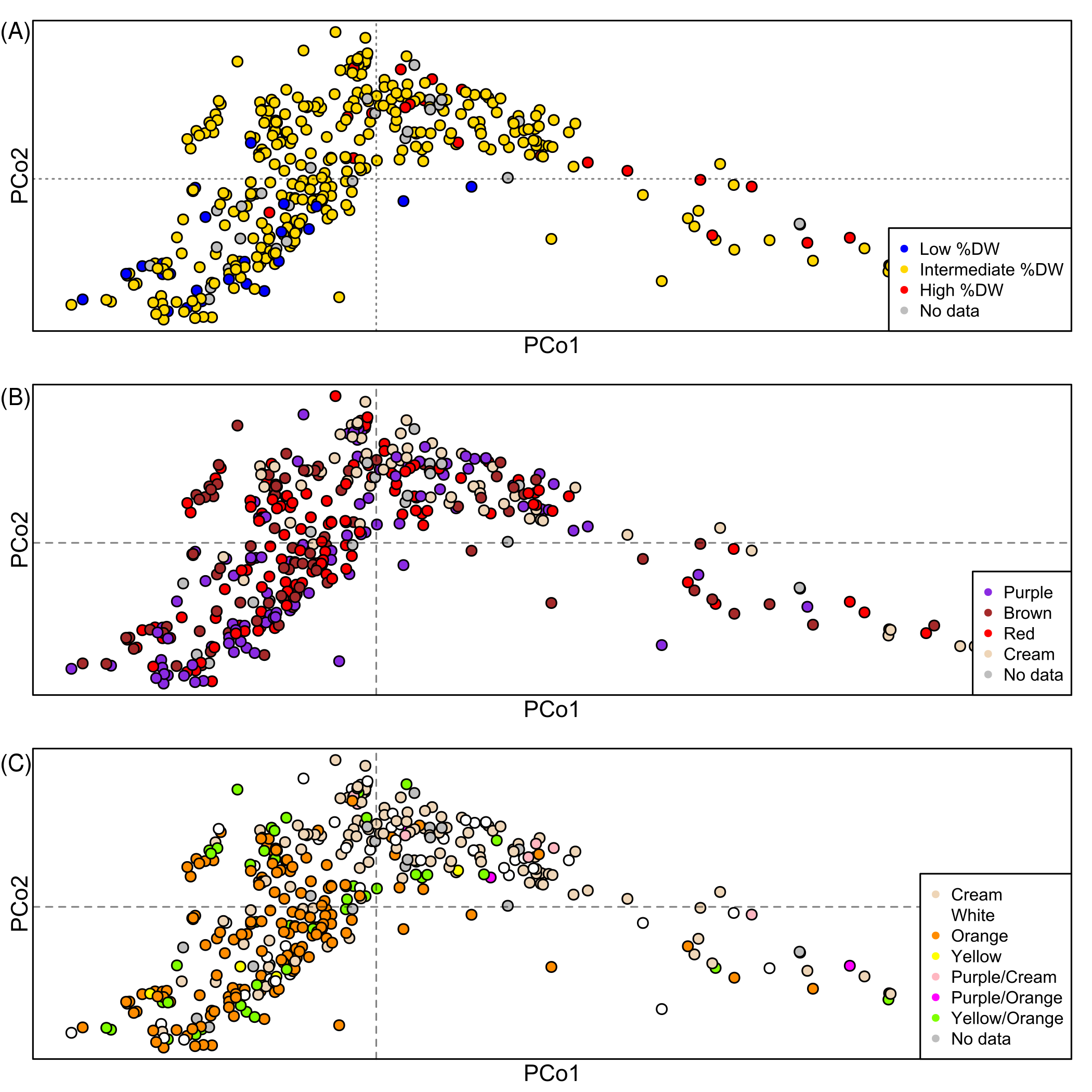

Supplement: FIGURE S5 — Linkage disequilibrium estimates (r2) of all genome-wide marker pairs plotted against corresponding interval between marker pairs. Curve (blue line) based on game smoothing method function shows distribution of all data points. Top and middle plot based on genotype data with allelic dosage information, while bottom plot is based on diploidized genotypes. [file Image_5.TIFF]

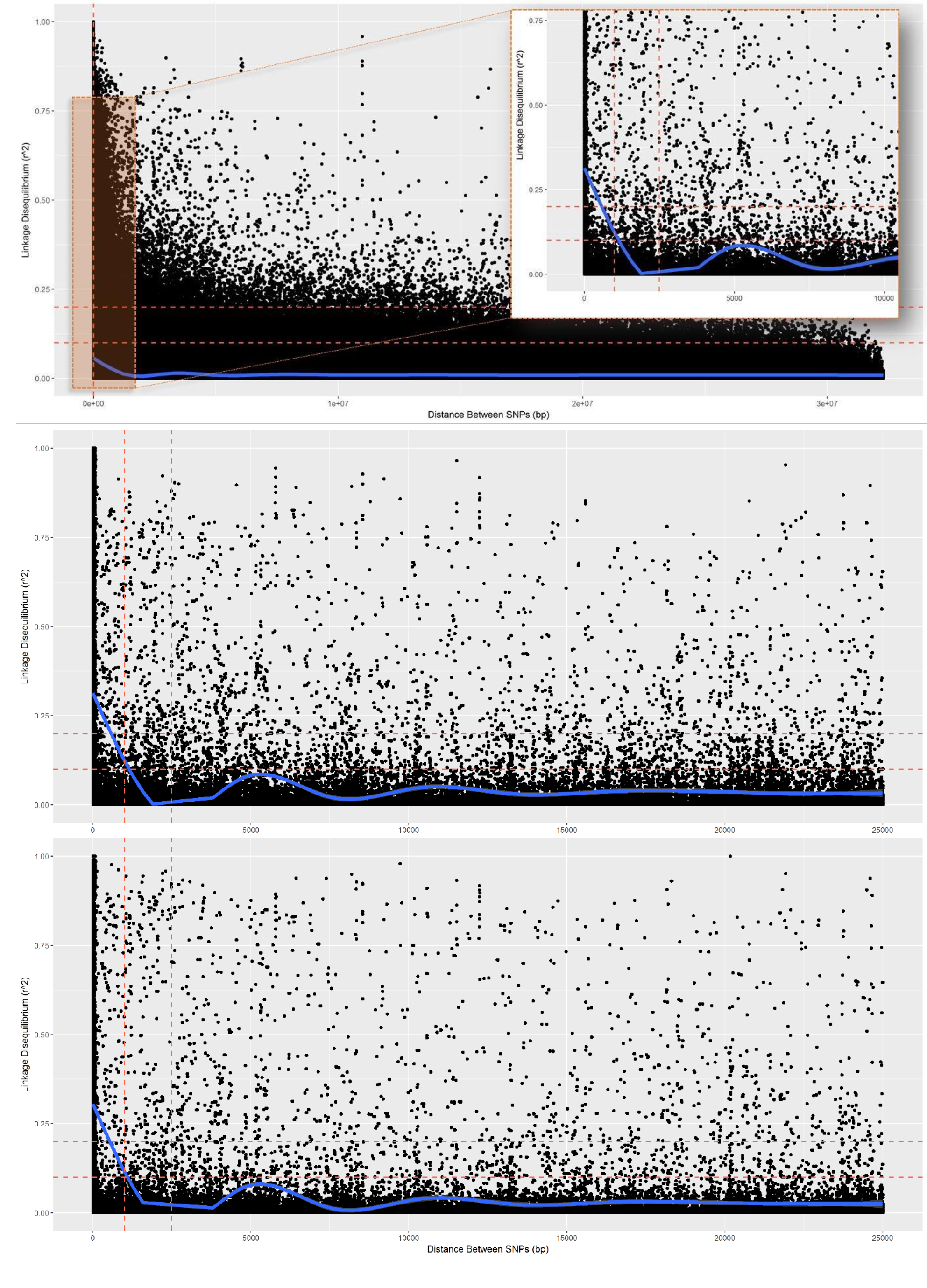

Supplement: Supplementary file 10 [file Image_6.TIF]
